# Identification of key genes and pathways between mild-moderate and severe asthmatics via bioinformatics analysis

**DOI:** 10.1038/s41598-022-06675-w

**Published:** 2022-02-15

**Authors:** Xiaolu Wu, Ran Li, Qu Xu, Feng Liu, Yue Jiang, Min Zhang, Meiling Tong

**Affiliations:** 1 Department of Child Health Care, Women’s Hospital of Nanjing Medical University (Nanjing Maternity and Child Health Care Hospital), Nanjing, China; 2 Shanghai Institute of Hematology, State Key Laboratory of Medical Genomics, National Research Center for Translational Medicine at Shanghai, Ruijin Hospital, Shanghai Jiao Tong University School of Medicine, Shanghai, China

**Keywords:** Biomarkers, Diseases

## Abstract

Severe asthma is the main reason for death and disability caused by asthma. However, effective biomarkers for severe asthma have not been identified. Here, we aimed to identify potential biomarkers in severe asthma. We identified 202 differentially expressed genes (DEGs) between severe asthma and mild-moderate asthma after integrating the results from GSE69683 and GSE27011 datasets. The enrichment analysis indicated that 202 DEGs were associated with metabolism- and immune-related processes. 10 hub genes were identified by Cytoscape and five of these genes’ AUC (area under the curve) values were greater than 0.6 in GSE69683. The AUC value reached to 0.701 when combined SEC61A1 and ALDH18A1 expression. The expression of the five hub genes was verified in an external dataset. The network analysis revealed that transcription factor (TF) WT1, ZEB1, RERE, FOSL1, and miR-20a may be involved in the development of asthma. In addition, we found cyclosporine and acetaminophen could interact with these hub genes and may be negatively associated with most of the five hub genes according to previous reports. Overall, key genes were identified between mild-moderate and severe asthmatics, which contributed to the understanding of the development of asthma.

## Introduction

Severe asthma is characterized by uncontrolled symptoms, airflow obstruction, and frequent exacerbations, which is the major reason for death and disability caused by asthma^1^. Mild-moderate to severe asthma is based on the level of treatment required to control symptoms or prevent asthma attacks^2^. Severe asthma affects only 3%–10% of the asthma population, but their health-care is estimated to contribute more than 60% of the cost with asthma^3^. It also imposes a considerable burden owing to poor symptoms, increased exacerbations, and medication side effects, which have profound effects on emotional and mental health, relationships, and careers^4^.

Substantial progress in understanding and treating type-2 asthma has been made in the past decades, including evidence-based biomarkers and the availability of novel targeted therapies^5^. However, more than 50% of patients with non-type-2 severe asthma remain poorly understood and treated^6^. The result of high-dose inhaled corticosteroids treatment in severe asthma patients is disappointing with uncontrolled symptoms and frequent exacerbations^7^. A greater mechanistic understanding of different biomarkers and phenotypes of severe asthma is an important step to personalized treatment, as well as being critical for discovering new efficacious therapies targeting non-type-2 pathways^8^.

The transcriptomic profiling in severe asthma has provided clues and insights on promising biomarkers and therapeutic targets^9^. A specific signature produced by transcriptome data of epithelial brushings could predict asthma patients’ response to inhaled corticosteroids (ICS) and associated with Th2-high inflammation^10,11^. In severe asthma, transcriptomic analyses demonstrated that novel biomarkers or phenotypes associated with clinical features^12^, adult-onset severe asthma^13^, Th2-high inflammation^9,14^, and corticosteroid insensitivity^15^. However, the underlying pathologic mechanism of several asthma is still unclear. In this study, we hypothesized key potential genes drive asthma progression and therefore, are associated with severe asthma. This study aimed to identify differentially expressed genes (DEGs) and potential mechanistic regulation networks between mild-moderate asthma and severe asthma.

## Methods

### Transcriptome data acquisition

We obtained microarray data for mild-moderate and severe asthma from Gene Expression Omnibus (GEO) database. The inclusive criteria are as follow: (1) Homo sapiens; (2) the number of sample size is more than 30; (3) complete information about the samples; (4) samples are blood source except for the validation dataset. At last, GSE69683 and GSE27011 were chosen for identifying potential biomarkers in the severe asthma; GSE76262 was considered as the validation dataset.

### Identification of DEGs

All data was normalized by log2(x + 1) transformed. The “limma” package^16^ from R software was used to identify DEGs between mild-moderate and severe asthma groups. Only genes’ false discovery rate (FDR) < 0.05 were considered as DEGs.

### Enrichment analysis

Gene set enrichment analysis (GSEA) analysis^17^ was performed using the GSEA software with the gene list of Gene Ontology (GO) gene sets (c5.all.v7.4.symbols.gmt). The "clusterProfiler" R package^18^ was used to perform GO and Kyoto Encyclopedia of Genes and Genomes (KEGG) analyses of these DEGs.

### Construction of the protein–protein interaction (PPI) network

Online tool STRING (https://string-db.org/) was used to construct PPI network based on the 202 DEGs. Cytoscape software^18^ (v3.7.2) was used to visualize the interaction results from the STRING. The cytoHubba plugin^19^ was used to obtain hub genes according to the degree method.

### Receiver operating characteristics (ROC) analysis

For ROC analysis of individual gene, outcome (mild-moderate or severe asthma) and individual gene expression were collected. Using "pROC" R package^20^, we calculated the AUC value. For ROC analysis of combined gene, outcome and each included gene expression were collected. Firstly, logistic regression was used to calculate the diagnostic score: β1 × gene1 expression + β2 × gene2 expression + β3 × gene3 expression… + βn × genen expression, where β was the correlation coefficient produced by the logistic regression analysis. Then, according to the outcome and diagnostic score, we calculated the AUC value using "pROC" R package. For the recommended model: diagnostic score = − 2.0892*SEC61A1 + (− 1.297)*ALDH18A1.

### Construction of transcription factor (TF)-gene and gene-miRNA networks

TF-gene and gene-miRNA networks were constructed using the online tool of NetworkAnalyst (https://www.networkanalyst.ca/). Based on the ENCODE ChIP-seq data (https://www.encodeproject.org/), we constructed the TF-gene network. According to miRTarBase v8.0 (https://mirtarbase.cuhk.edu.cn/php/download.php), we constructed the gene-miRNA network. We extracted expression of TFs and hub genes from GSE69683. Spearman correlation analysis were used to explore the association between hub genes and TFs. "ggplot2" R package (https://ggplot2.tidyverse.org/) was used to visualize the results.

### Prediction of interacted chemicals

According to the data from Comparative Toxicogenomics Database (CTD) (http://ctdbase.org/), we constructed the protein-chemical network using the NetworkAnalyst tool.

### Statistics analysis

Statistical analyses of all data were performed by R software (version 4.1.1, https://www.r-project.org/). Student’s t-test was used to compare the differences between the two groups. P < 0.05 was considered statistically significant.

## Results

### Identification of DEGs

**Figure 1:**
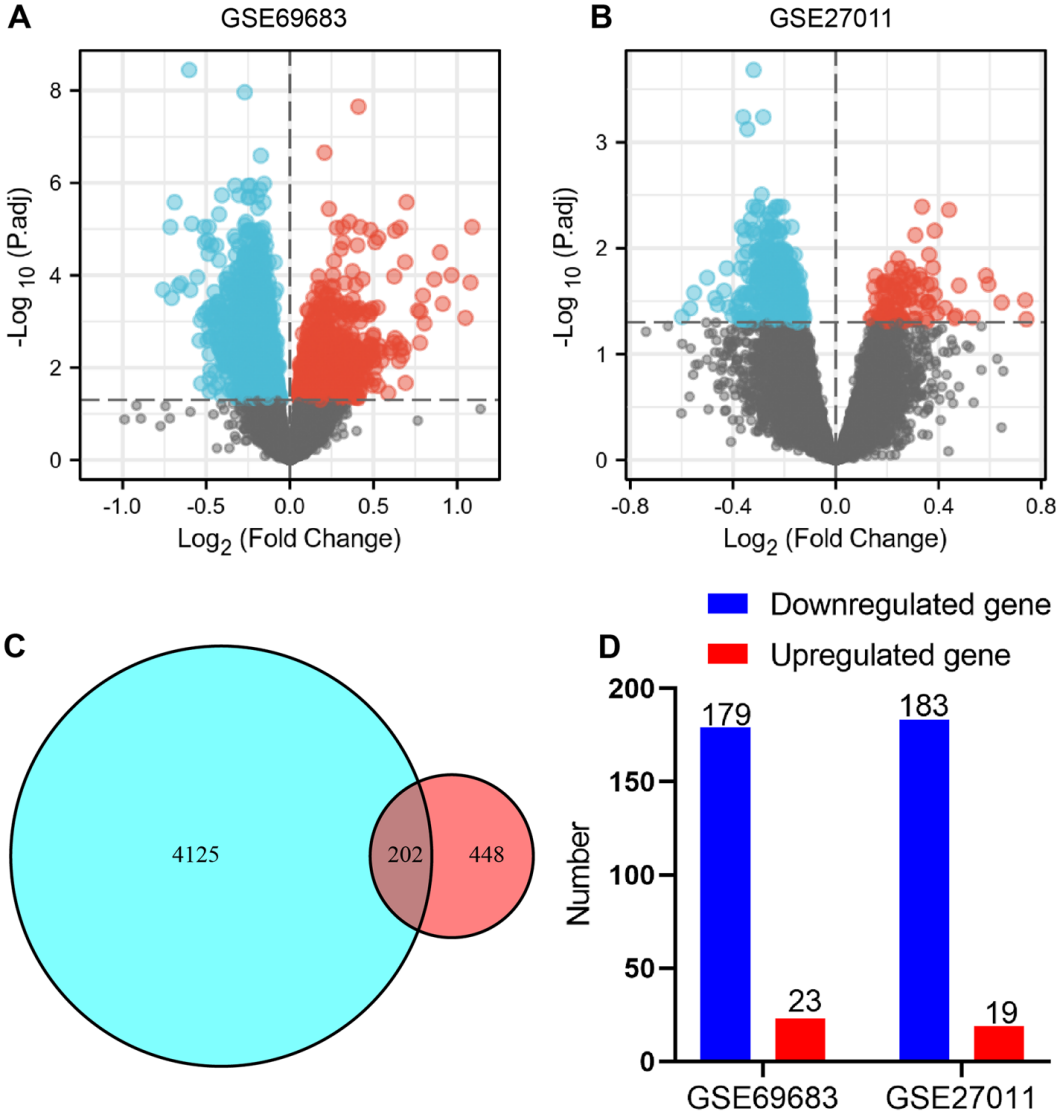
Identification of DEGs. The volcano plot for GSE69683 (**A**) and GSE27011 (**B**). Red dots represent upregulated genes; blue dots represent downregulated genes; grey dots represent genes with no significant expression differences. (**C**) Venn diagram for GSE69683 (the blue set) and GSE27011 (the red set). (**D**) The number of upregulated and downregulated genes of shared 202 genes in GSE69683 and GSE27011, respectively.

4327 DEGs were identified between severe and mild-moderate asthma in GSE69683, including 2575 downregulated genes and 1752 upregulated genes (Fig. 1A). 650 DEGs were identified between severe and mild-moderate asthma in GSE27011, including 548 downregulated genes and 102 upregulated genes (Fig. 1B). Taking the intersection of the results from GSE69683 and GSE27011, we obtained 202 genes for further explorations (Fig. 1C). Among 202 genes, 23 genes were upregulated and 179 genes were downregulated in GSE69683; 19 genes were upregulated and 183 genes were downregulated in GSE27011 (Fig. 1D). In addition, 8 of 202 genes was found upregulated in one study and downregulated in the other, including FFAR3, ANKRD11, SLC39A14, ZC3H7B, TMEM107, RPUSD3, NDUFA4, and CTBP1-AS2. We consider that two independent datasets presenting contrary expression differences in 8 of 202 genes is acceptable.

### Enrichment analysis

**Figure 2:**
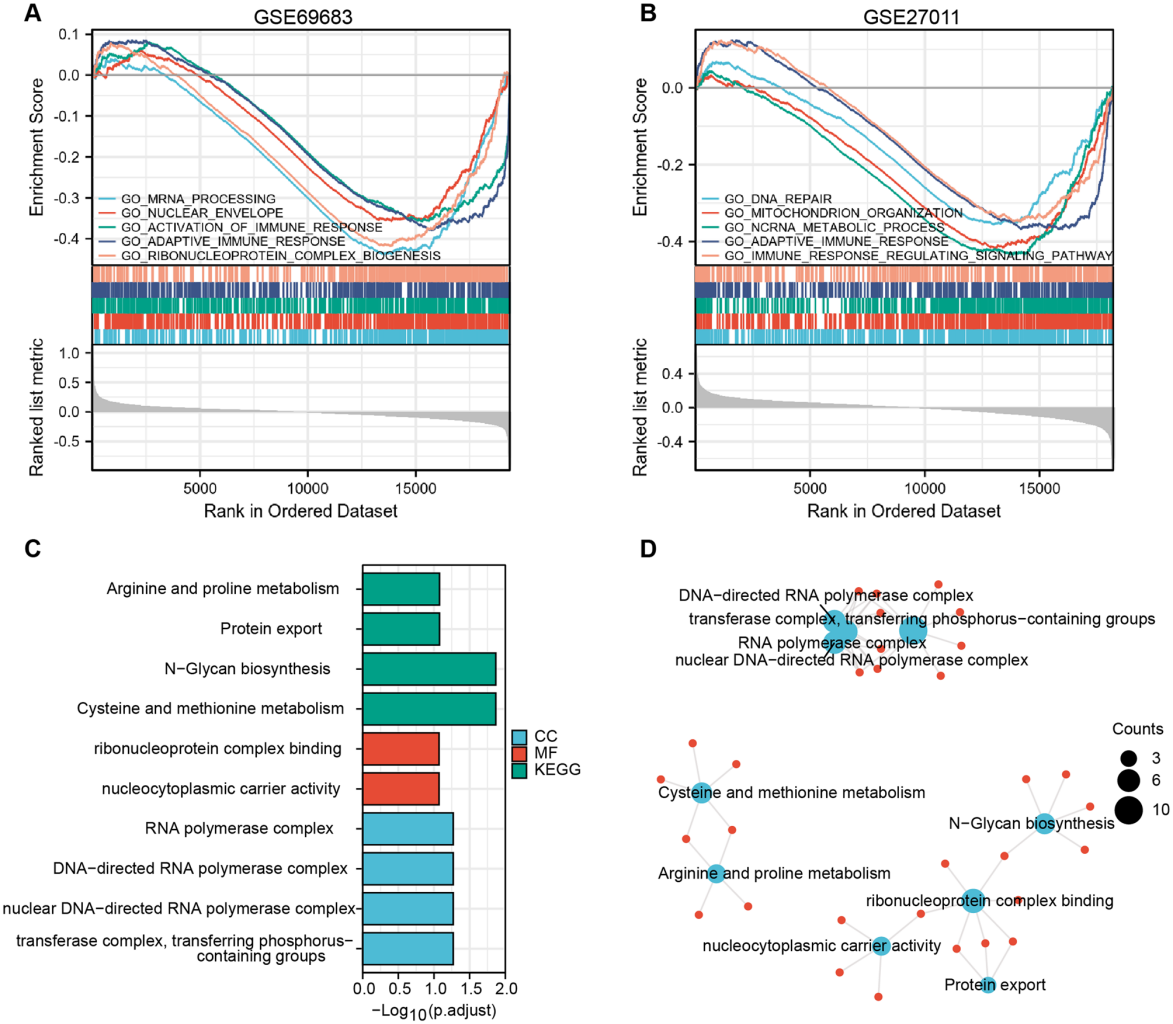
Enrichment analysis. GSEA analysis for GSE69683 (**A**) and GSE27011 (**B**). The results include upper, middle, and lower parts. The upper part: the peak in the right represents core molecules were mainly concentrated in the mild-moderate asthma group; the middle part: each vertical line represents a molecule in the gene set; the lower part: visualization of values given by the uploaded normalized data. GO and KEGG analysis for DEGs displayed in the form of histogram (**C**) and the network diagram (**D**).

GSEA analysis for all genes ranked by log_2_Fold Change showed that the nucleic acid metabolism-related and immune-related process were downregulated in severe asthma in both GSE69683 and GSE27011 datasets (Fig. 2A,B). The enrichment analysis of shared 202 genes indicated that arginine and proline metabolism, protein export, N-Glycan biosynthesis, cysteine and methionine metabolism and nucleic acid complex were associated with the progression of asthma (Fig. 2C,D).

### PPI network analysis

**Figure 3:**
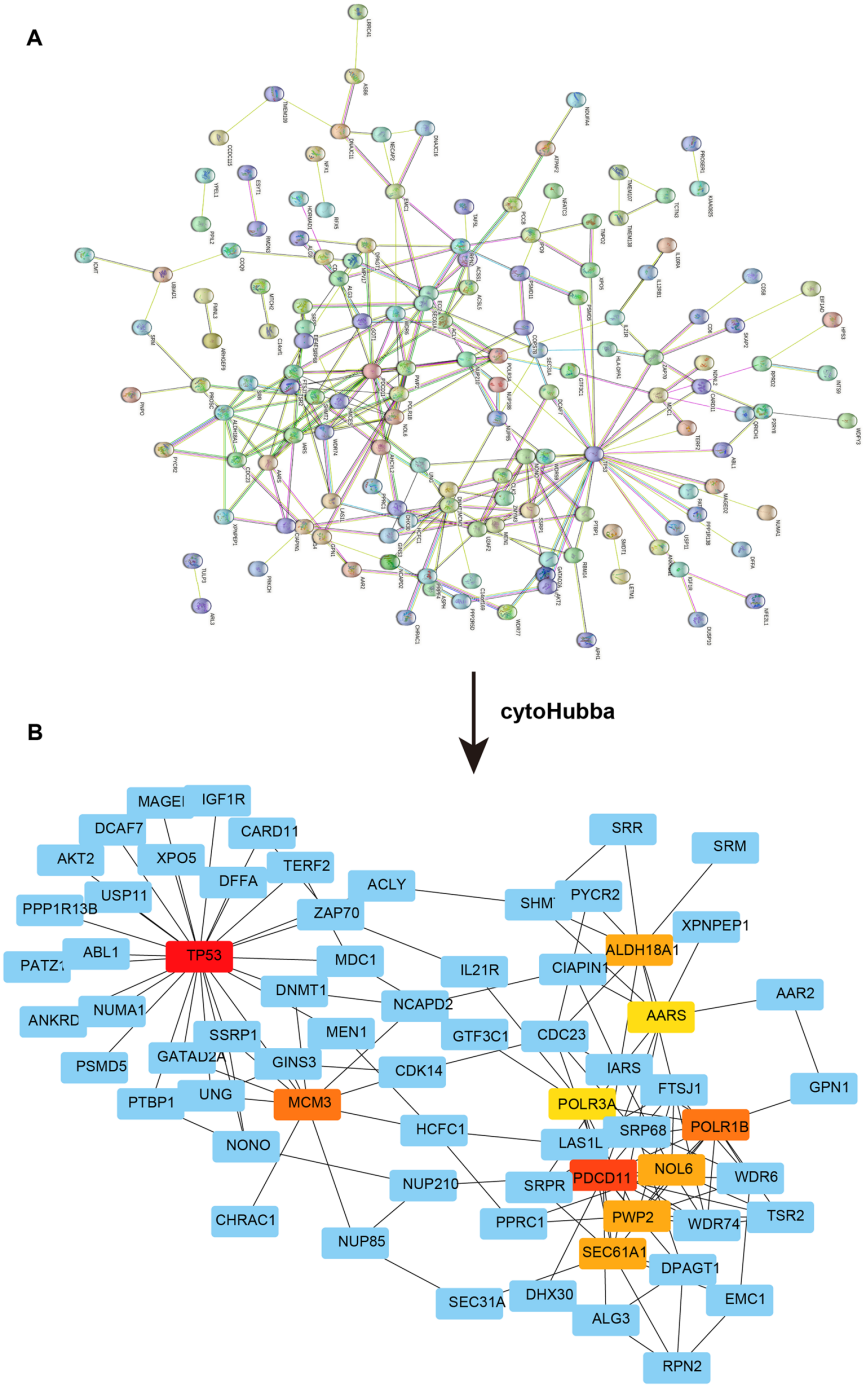
PPI network analysis. (**A**) PPI analysis for all DEGs. Network nodes represent proteins. Edges represent protein–protein associations. Light blue and purple edges mean known interactions; Dark blue, green, and red edges mean predicted interactions. (**B**) The network of 10 hub genes determined by the cytoHubba. The darker the color, the higher the degree value.

Next, we constructed PPI network of the 202 genes to understand the interactions between these functional proteins (Fig. 3A). Then, the cytoHubba plugin was used to identify hub genes in this network and we obtained 10 hub genes (Fig. 3B). The detailed information of 10 hub genes was shown in Table 1.

**Table 1:** 10 hub genes.

Gene symbol	Description	Degree
TP53	Tumor protein p53	52
PDCD11	Programmed cell death 11	32
POLR1B	RNA polymerase I subunit B	22
MCM3	Minichromosome maintenance complex component 3	22
SEC61A1	Sec61 translocon alpha 1 subunit	18
PWP2	PWP2 periodic tryptophan protein homolog (yeast)	18
NOL6	Nucleolar protein 6	18
ALDH18A1	Aldehyde dehydrogenase 18 family member A1	18
POLR3A	RNA polymerase III subunit A	16
AARS	Alanyl-tRNA synthetase	16

### ROC curve of 10 hub genes in mild-moderate and severe asthma samples

**Figure 4:**
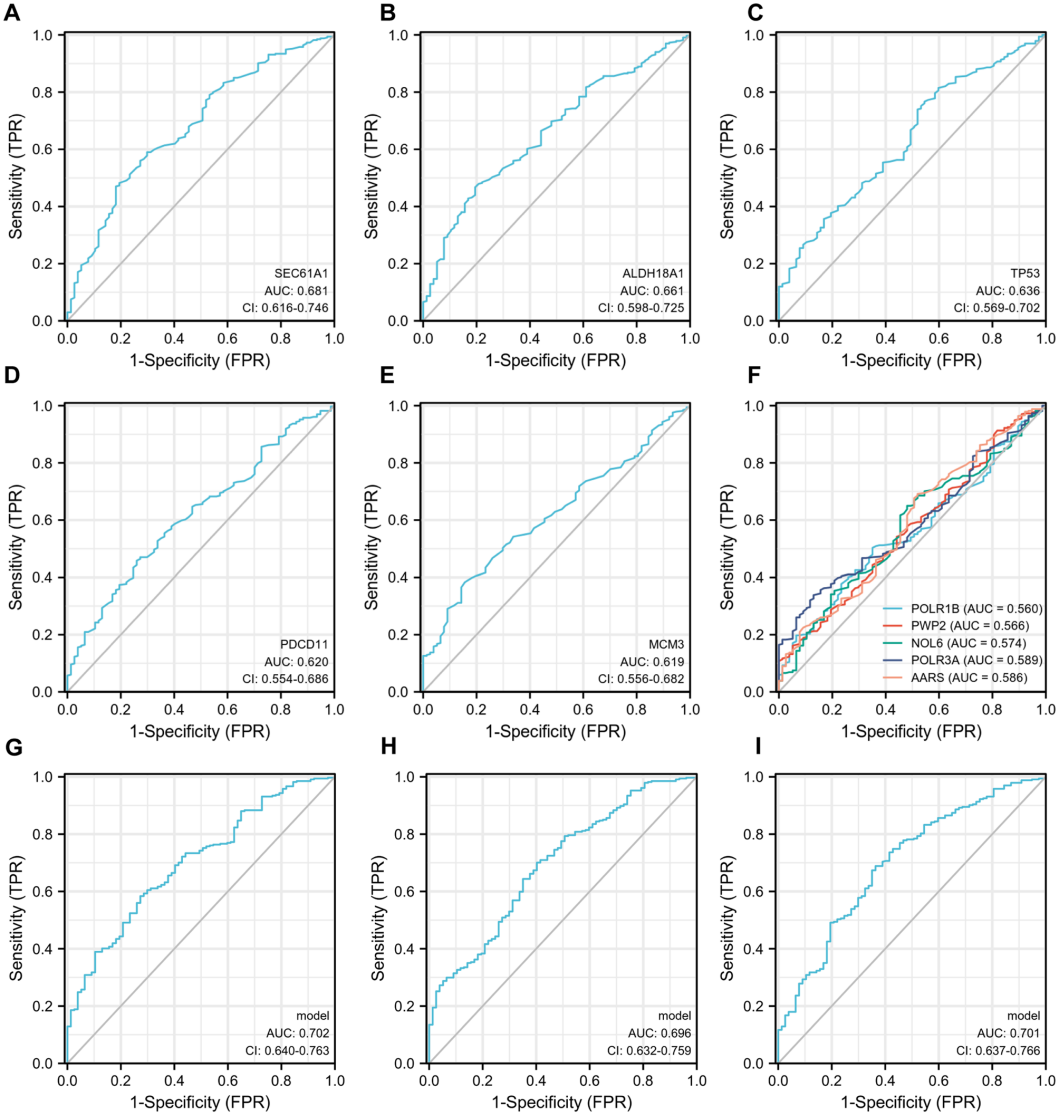
Evaluation of the hub genes. ROC (Receiver operating characteristic) curves for SEC61A1 (**A**), ALDH18A1 (**B**), TP53 (**C**), PDCD11 (**D**), MCM3 (**E**), the other five genes (**F**), combined 10 hub genes (**G**), combined AUC top 5 genes (**H**), and combined AUC top 2 genes (**I**). *AUC* area under the curve, *CI* Confidence interval.

In GSE69683, roc curve was used to evaluate 10 hub genes’ distinguishing ability between mild- moderate and severe asthma. The AUC value (> 0.6) of hub genes are as follows: SEC61A1 (0.681, Fig. 4A), ALDH18A1 (0.661, Fig. 4B), TP53 (0.636, Fig. 4C), PDCD11 (0.620, Fig. 4D), MCM3 (0.619, Fig. 4E). The AUC value of other five genes was between 0.5 and 0.6 (Fig. 4F). The five hub genes with AUC value > 0.6 were chosen for a further investigation. Since each single gene with AUC value < 0.7, we tried to combine these genes to increase the AUC value. The results showed that AUC values were 0.702 (combined 10 hub genes, Fig. 4G), 0.696 (combined AUC top 5 genes, Fig. 4H), and 0.701 (combined AUC top 2 genes, Fig. 4I), respectively. Considering the reliability and cost, we recommended combined SEC61A1 and ALDH18A1 genes as a diagnostic model.

### Verification of the hub gene expression in the external dataset

**Figure 5:**
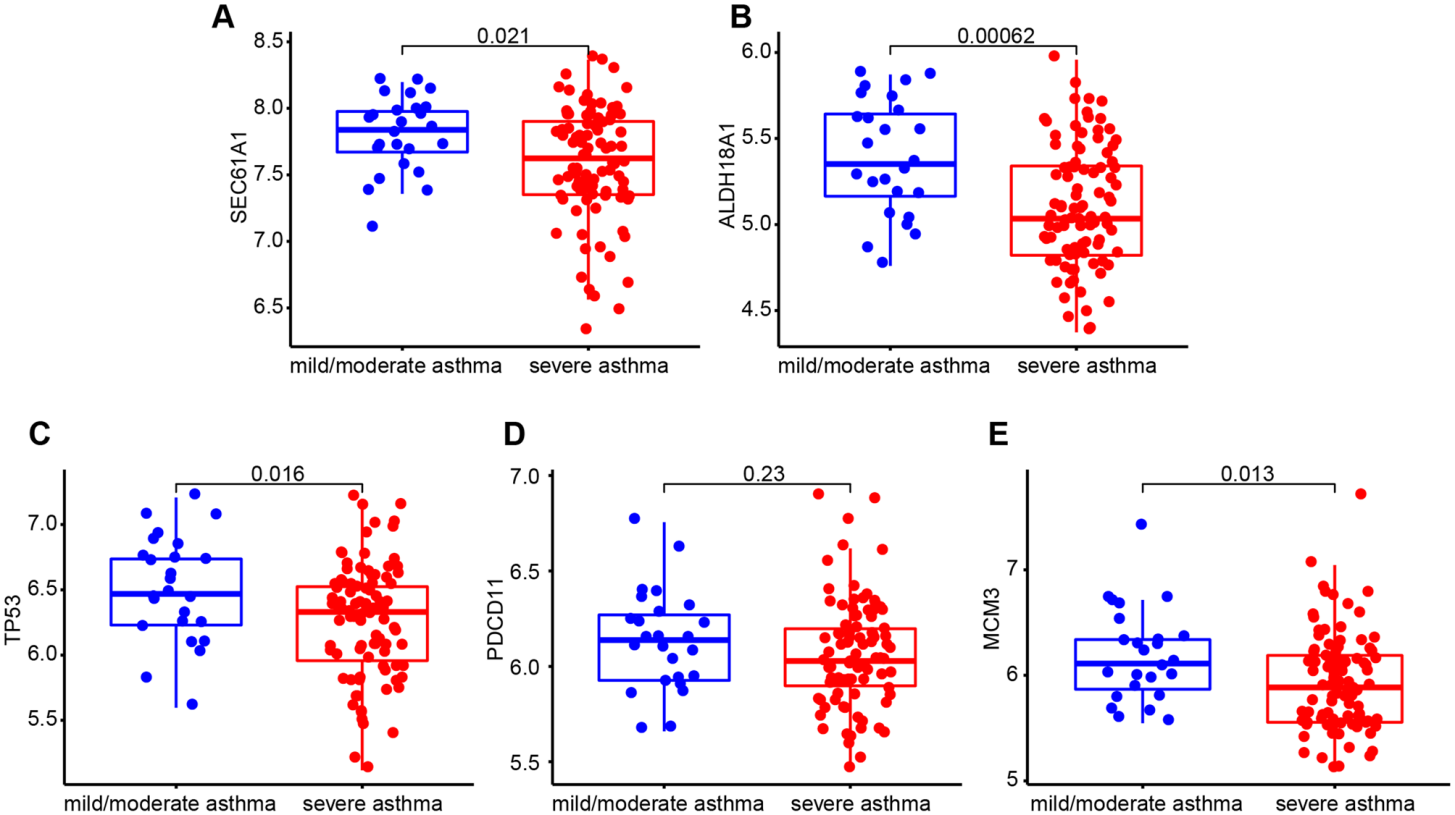
Verification of the hub gene. The gene expression between mild-moderate asthma (Blue) and severe asthma (Red), including SEC61A1 (**A**), ALDH18A1 (**B**), TP53 (**C**), PDCD11 (**D**), and MCM3 (**E**).

To confirm whether these hub genes were meaningful in the progression of asthma, we identified their expression in the external dataset (GSE76262, data produced from sputum samples). We consider that if results from different tissues are consistent, this may be more convincing. The results showed that SEC61A1 (Fig. 5A), ALDH18A1 (Fig. 5B), TP53 (Fig. 5C), PDCD11 (Fig. 5D) and MCM3 (Fig. 5E) were lowly expressed in severe asthma, which was consistent with the results in GSE69683 and GSE27011.

### TF-gene interactions and gene-miRNA networks

**Figure 6:**
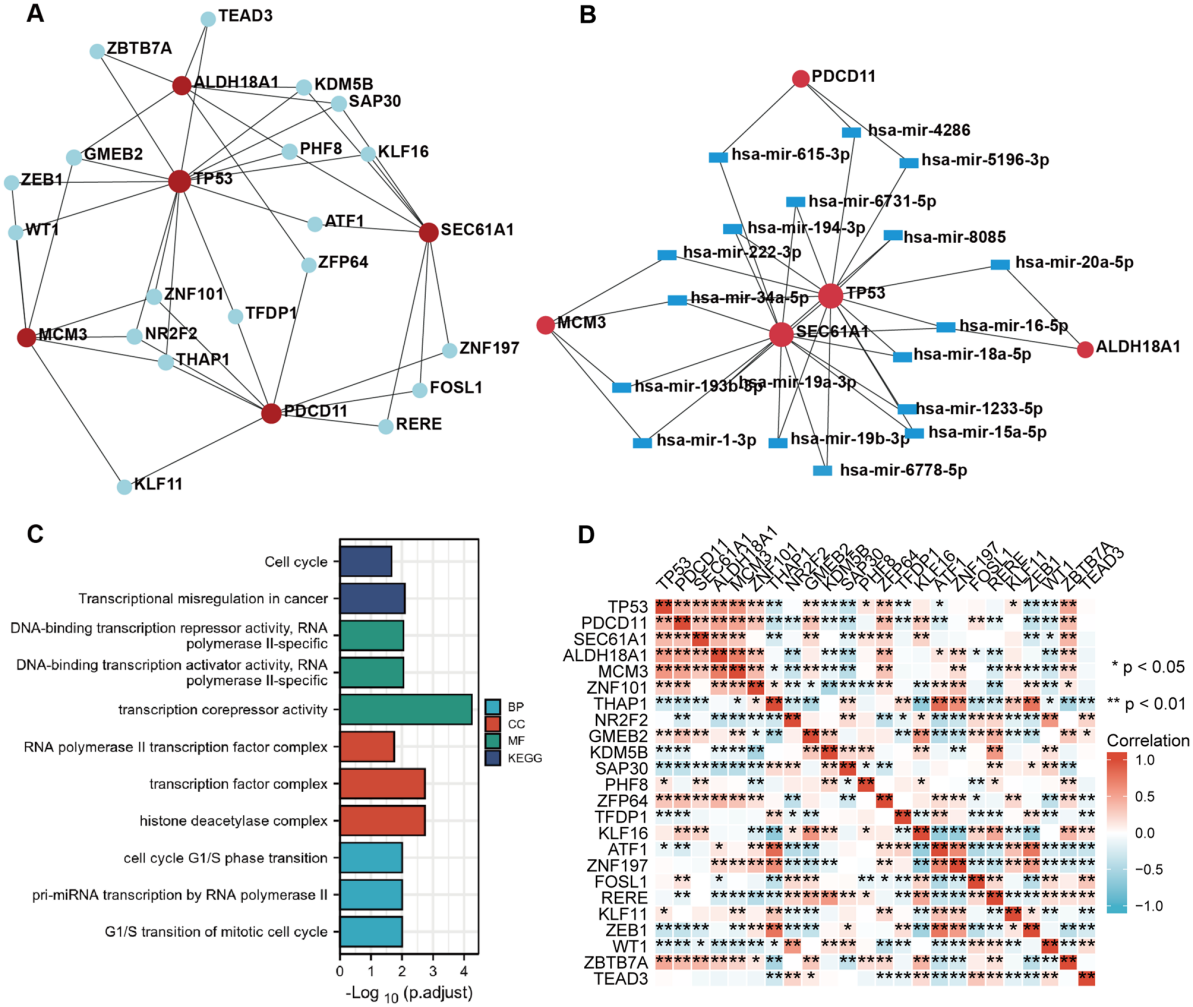
TF-gene interactions and gene-miRNA networks. (**A**) The TF-gene interaction network. Red spots represent hub genes; blue spots represent TFs. (**B**) The gene-miRNA network. Red spots represent hub genes; blue rectangles represent miRNAs. (**C**) GO and KEGG analysis for the TF-gene interaction network displayed in the form of histogram. (**D**) Heatmap of expression correlation in severe asthmatics from GSE69683. Five hub genes and TFs in the network were analyzed using Spearman method.

To understand the potential regulation of hub genes, we performed a comprehensive network analysis, including TF and miRNA. The TF-gene interaction network consists of 24 nodes and 45 edges (Fig. 6A). The gene-miRNA network includes 23 nodes and 38 edges (Fig. 6B). Based on the hub genes and predicted TFs, enrichment analysis showed that these genes were associated with cell cycle, transcriptional misregulation and histone deacetylase complex (Fig. 6C). To further understand the relation of activation or repression of TFs with the hub genes, we conducted a correlation analysis in GSE69683. Interestingly, asthma-related TFs, including WT1, ZEB1, and RERE, were negatively associated with most of hub genes (Fig. 6D).

### Protein–chemical interactions

**Figure 7:**
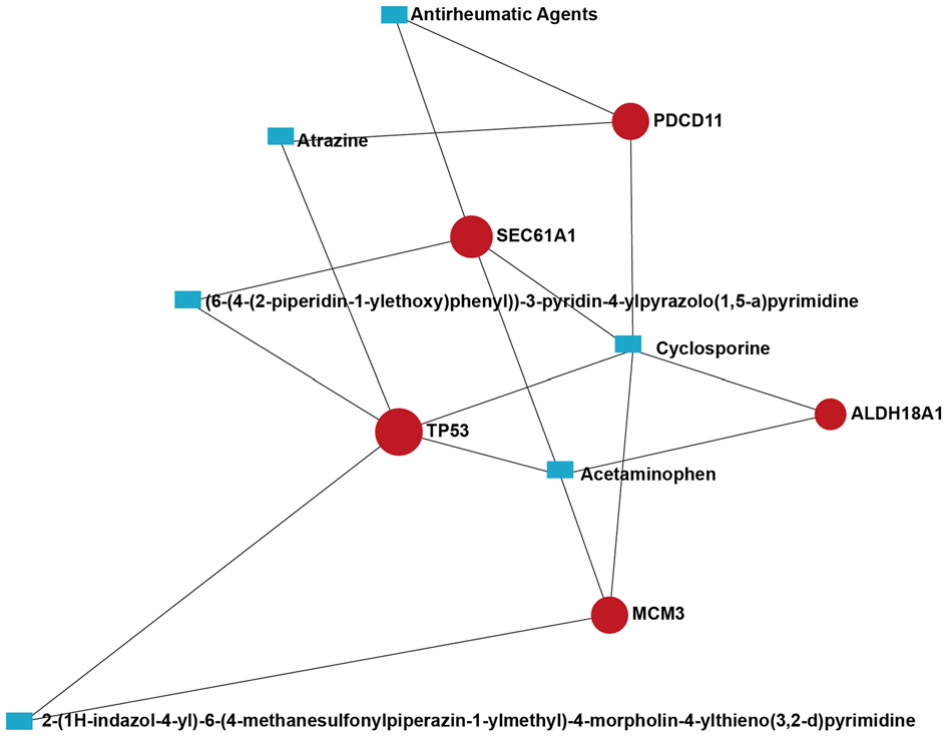
Protein-chemical interactions. Red spots represent hub genes; blue rectangles represent chemicals.

We consider the hub genes could be potential therapeutic targets for severe asthma patients, so we constructed a network between five hub genes and chemicals. The results showed that cyclosporine can interact with all five genes and acetaminophen can react to four genes. The other four chemicals only interact with two genes (Fig. 7).

## Discussion

Identification of novel biomarkers can contribute to the patient classification, therapy response, and clinical outcome prediction^21^. In asthma, most identified biomarkers are limited to the Th2 phenotype, and no effective biomarkers have been verified for severe asthma^22^. Blood samples serve as an alternative to the challenging and costly approach of obtaining airways samples in severe asthma^23^. In this study, we identified five potential biomarkers in severe asthma based on blood samples. TF-gene interactions and gene-miRNA networks associated with the five biomarkers were constructed. In addition, we found two chemicals that have an ability to interact with the identified biomarkers.

Nucleic acid metabolism-related pathways are associated with multiple biological processes, including DNA repair^24^, chronic inflammation^25^, collagen deposition^26^, and so on. Our data showed that dysregulated nucleic acid metabolism-related pathways were involved in the asthma progression. Some cytokines were considered to play important roles in asthma progression. Specifically, Eosinophil-associated fibrogenic factors (such as TGF-β) can lead to airway remodeling characterized by smooth muscle thickening, goblet cell metaplasia, and extracellular matrix protein deposition^27^. In addition, increased IL-1β and IL-17 also were considered with asthma disease severity^28^. Meanwhile, TGF-β, IL-1β, and IL-17 were considered as immunosuppressive factors^29^. Our GSEA results indicated immune-related processes were downregulated in severe asthma. We considered that chronic inflammation in lung may induce an immunosuppressive microenvironment. A typical example is that chronic inflammation promotes cancer progression through immune evasion^29^. However, immune status is a complex and dynamic process, whether severe asthmatics showing an immunosuppressive microenvironment compared with mild-moderate asthmatics is needed to be further explored.

To date, the five genes in this study have not been reported in severe asthma. Minichromosome maintenance 3 protein (MCM3) protein is a member of 6 highly conserved minichromosome maintenance proteins family (MCM2-MCM7) that bind to replication origins and permit them to maintain a single round of DNA replication. Even a minor reduction of the MCM expression destabilizes the genome and predisposes to increased incidence of diseases^30^. Previous studies focused on the role of MCM3 in tumor development and growth^31^ and there is no study about the relationship between MCM3 and asthma. We found MCM3 was lowly expressed in severe asthma, which indicated patients with severe asthma may suffered with the disorder of DNA replication and cell cycle. The transcription factor encoded by TP53 gene is a crucial hurdle to carcinogenesis and inactivation of TP53 is the most common mutation in human cancers^32^. Moreover, TP53 methylation is a suitable blood biomarker to predict late-onset asthma (LOA)^33^. PDCD11 (Programmed Cell Death 11) is a NF-kappa-B-binding protein that colocalizes with U3 RNA in the nucleolus and is required for rRNA maturation and generation of 18S rRNA^34^. Rare studies reported the function of PDCD11 in diseases and this gene is needed to be explored in the future. Aldehyde dehydrogenase 18 Family member A1 (ALDH18A1) is a NAD(P)H- and ATP-dependent enzyme that can convert glutamate to pyrroline-5-carboxylate (P5C), an important intermediate step in proline metabolism. ALDH18A1 is associated with poor prognosis in patients with hepatocellular carcinoma^35^ and breast cancer^36^. ALDH18A1 and glutaminase protein are highly expressed in proliferative estrogen receptor positive (ER^+^) tumor cells compared to ER^−^ cells^36^. Increasing evidence has convincingly demonstrated that high expression of ALDH18A1 is a poor prognosis factor in different tumor types and increased proline biosynthesis is needed in cancer cells^37^. SEC61 translocon subunit alpha 1 (SEC61A1) is the main subunit of the SEC61 complex that plays a role in the main polypeptide conduction pathway of the endoplasmic reticulum membrane. Missense mutation of SEC61A1 can induce immune-related diseases, such as plasma cell deficiency^38^. We found PDCD11 was negatively associated with asthma-related TFs, including WT1, ZEB1, and RERE; ALDH18A1 was negatively associated with WT1 and RERE; SEC61A1 was negatively related with WT1 and ZEB1. Hence, PDCD11, ALDH18A1, and SEC61A1 may play a role in the development of asthma through transcriptional regulation-related mechanisms.

To elucidate the potential mechanism of the five genes in severe asthma, we conducted a TF-gene and gene-miRNA interaction analysis. The identified TFs and miRNAs may be involved in the pathological process of severe asthma. Wilms tumor 1 (WT1) is a suppressor of MMP-9, regulated by a NO-mediated signaling pathway in human lung epithelial cells. MMP-9 expression correlates with the severity of asthma and chronic obstructive pulmonary disease. Zinc finger E-box binding homeobox 1 (ZEB1), an epithelial-mesenchymal transition (EMT) key regulator, has been reported involved in the airway smooth muscle cell proliferation^39^ and nickel exposure-induced impairment of human lung epithelial cells^40^. Through transcriptome-wide association studies, transcription factor RERE was identified as a candidate causal gene of asthma^41^. The whole-genome expression analysis of peripheral blood mononuclear cells indicated that FOSL1 was positively associated with aspirin-intolerant asthma^42^. In addition, miR-20a epigenetically regulated the IL-10 production by targeting HDAC4 and attenuated allergic inflammation in the human mast cells. Hence, the identified hub genes, TFs and miRNAs may contribute to the progression of asthma.

Through the protein-chemical interaction analysis, we identified cyclosporine and acetaminophen may have effects on severe asthma. Cyclosporin, as an immunosuppressive agent, has benefits in the treatment of inflammatory disorders. Patients with chronic severe asthma are dependent on the oral corticosteroids and alternative therapies are needed to reduce the use of corticosteroids. Cyclosporin has been considered as a potential useful agent in the treatment of chronic severe asthma^43,44^. Moreover, for lung transplant recipients, improvements in lung function were observed in the group receiving inhaled cyclosporine^45^. Acetaminophen (paracetamol) is widely used in infants and toddlers as the drug of choice for fever for its recognized safety for most children. However, the findings of a case–control study raised a concern about the safety of acetaminophen that indicated frequent use of acetaminophen was associated with the occurrence and development asthma. This concern led to decades of debate about whether frequent acetaminophen use leads to asthma in children who would otherwise be asthma-free and worsens asthma in patients who already have asthma^46^. Until now, for pediatricians the question whether “to give or not to give” acetaminophen might be a bitter pill to swallow because ambiguous conclusions about the question. According to the Comparative Toxicogenomics Database and literature review, we found Cyclosporine results in increased expression of TP53 mRNA^47,48^, increased expression of SEC61A1 mRNA^49–51^, increased expression of PDCD11 mRNA^50^, decreased expression of ALDH18A1 mRNA^50,52^, and decreased expression of MCM3 mRNA^48,50,53^. Moreover, Acetaminophen results in increased expression of SEC61A1 mRNA^54,55^, increased activity of TP53 protein^56^, increased expression of TP53 mRNA^57,58^, increased expression of MCM3 mRNA^55^, and decreased expression of ALDH18A1 mRNA^59,60^. Although Cyclosporine and Acetaminophen were reported inhibition the expression of most of five hub genes, the proof were produced from non-asthma population. A correlation with the clinical and complex etiopathogenetic mechanisms of asthma is necessary. In the era of precision medicine, it is important to use specific drugs for increasingly specific asthmatic phenotypes.

## Conclusion

We identified 202 DEGs between mild-moderate and severe asthmatics from GSE69683 and GSE27011. The results of GSEA analysis indicated that nucleic acid metabolism-related and immune-related process were downregulated in severe asthma. 10 hub genes were identified using cytoHubba plugin and five of these genes’ AUC values were greater than 0.6. In addition, a diagnostic model combined SEC61A1 and ALDH18A1 genes could effectively distinguish mild-moderate from severe asthmatics (AUC value: 0.701). Finally, cyclosporine and acetaminophen were able to interact with most of the five hub genes. Collectively, we identified key genes and potential pathways between mild-moderate and severe asthmatics and provided clues for the mechanisms of the development of asthma at the transcriptome level.

## Data Availability

The data can be acquired in GEO database (https://www.ncbi.nlm.nih.gov/geo/).
